# 3D domain swapping in the TIM barrel of the α subunit of *Streptococcus pneumoniae* tryptophan synthase

**DOI:** 10.1107/S2059798320000212

**Published:** 2020-01-31

**Authors:** Karolina Michalska, Marcin Kowiel, Lance Bigelow, Michael Endres, Miroslaw Gilski, Mariusz Jaskolski, Andrzej Joachimiak

**Affiliations:** a Midwest Center for Structural Genomics, X-ray Science Division, Argonne National Laboratory, Argonne, IL 60439, USA; b Center for Structural Genomics of Infectious Diseases, Consortium for Advanced Science and Engineering, University of Chicago, Chicago, IL 60637, USA; c Structural Biology Center, X-ray Science Division, Argonne National Laboratory, Argonne, IL 60439, USA; d Center for Biocrystallographic Research, Institute of Bioorganic Chemistry, Polish Academy of Sciences, Poznan, Poland; e Department of Crystallography, Faculty of Chemistry, A. Mickiewicz University, Poznan, Poland; f Department of Biochemistry and Molecular Biology, University of Chicago, Chicago, IL 60637, USA

**Keywords:** tryptophan synthase, TIM barrel, 3D domain swapping, protein oligomerization, *Streptococcus pneumoniae*

## Abstract

Tryptophan synthase catalyzes the last two steps of tryptophan biosynthesis in plants, fungi and bacteria. It consists of two protein chains, designated α and β, encoded by *trpA* and *trpB* genes, that function as an αββα complex. Structural and functional features of tryptophan synthase have been extensively studied, explaining the roles of individual residues in the two active sites in catalysis and allosteric regulation. TrpA serves as a model for protein-folding studies. In 1969, Jackson and Yanofsky observed that the typically monomeric TrpA forms a small population of dimers. Dimerization was postulated to take place through an exchange of structural elements of the monomeric chains, a phenomenon later termed 3D domain swapping. The structural details of the TrpA dimer have remained unknown. Here, the crystal structure of the *Streptococcus pneumoniae* TrpA homodimer is reported, demonstrating 3D domain swapping in a TIM-barrel fold for the first time. The N-terminal domain comprising the H0–S1–H1–S2 elements is exchanged, while the hinge region corresponds to loop L2 linking strand S2 to helix H2′. The structural elements S2 and L2 carry the catalytic residues Glu52 and Asp63. As the S2 element is part of the swapped domain, the architecture of the catalytic apparatus in the dimer is recreated from two protein chains. The homodimer interface overlaps with the α–β interface of the tryptophan synthase αββα heterotetramer, suggesting that the 3D domain-swapped dimer cannot form a complex with the β subunit. In the crystal, the dimers assemble into a decamer comprising two pentameric rings.

## Abstract 2

The structure of *Streptococcus pneumoniae* TrpA, which is the α subunit of tryptophan synthase, reveals 3D domain swapping, which has never before been observed for a TIM-barrel protein.

## 1. Introduction   

Tryptophan synthase is a key enzyme in l-tryptophan biosynthesis in plants, fungi and bacteria. The enzyme is composed of two catalytic units, TrpA (α subunit) and TrpB (β subunit), that associate into a functional heterotetrameric αββα hydrolyase complex (TrpAB) (Dunn, 2012 ▸; Dunn *et al.*, 2008 ▸; Raboni *et al.*, 2003 ▸, 2009 ▸). The enzyme catalyzes two reactions: the α subunit converts 1-*C*-(indol-3-yl)glycerol 3-phosphate (IGP) to indole and d-glyceraldehyde 3-phosphate, while the β subunit condenses indole with l-serine to give l-tryptophan, with pyridoxal 5′-phosphate (PLP) functioning as a cofactor (Buller *et al.*, 2016 ▸). In this process, the indole molecule is passed from subunit α to the active site of subunit β via an ∼25 Å tunnel to avoid indole leaking through the cell membranes (Hyde *et al.*, 1988 ▸). The two subunits are enzymatically active when separated, but within the heterotetrameric complex their efficiencies are much greater owing to cooperativity. The mutual allosteric activation of the two subunits, which occurs through a set of specific interactions, represents one of several layers of regulation in the energetically costly tryptophan biosynthesis. Additional controls are in place at the transcription and metabolic levels to ensure fast response and efficient resource utilization (Dunn, 2012 ▸; Dunn *et al.*, 2008 ▸; Houben & Dunn, 1990 ▸; Joachimiak *et al.*, 1983 ▸).

The function, structure, folding and dynamics of TrpA, both alone and within the αββα complex, have been studied extensively over the past 60 years using *Escherichia coli* and *Salmonella typhimurium* model systems (Bonner & Yanofsky, 1951 ▸; Henning *et al.*, 1962 ▸). The *Streptococcus pneumoniae* subunit α (*Sp*TrpA) studied in the present work is a 258-residue protein which in its monomeric form assumes the canonical (β/α)_8_ TIM-barrel fold with three additional helices (Supplementary Fig. S1; Michalska *et al.*, 2019 ▸). The TIM barrel represents one of the most abundant and versatile protein folds in nature, and is found in all kingdoms of life and in viruses. It has evolved to serve a wide variety of enzyme functions including hydrolase, transferase and lyase functions, amongst others (Nagano *et al.*, 2002 ▸). Structural analyses suggest that TIM-barrel proteins evolved from a common half-barrel ancestor by gene duplication and fusion (Alva & Lupas, 2018 ▸; Höcker, 2014 ▸). More specifically, TrpA is thought to have arisen from a common TIM-barrel ancestor that contained a structurally conserved phosphate-binding motif. Nicolet and Drennan reported a 3/4-barrel and predicted a half-barrel structure (Nicolet & Drennan, 2004 ▸). The TIM barrel has been shown to be related to proteins with the flavodoxin-like fold (Farías-Rico *et al.*, 2014 ▸).

Proteolytic studies of TrpA from *E. coli* have shown that both the N-terminal (residues 1–188) and C-terminal (189–268) fragments can be isolated independently and that mixing the two restores the activity of the α subunit. This is consistent with observations that TIM-barrel enzymes subjected to fragment complementation and circular permutation tolerate such modifications, and further confirms that this fold is composed of two separate N- and C-terminal modules (Hiraga *et al.*, 2004 ▸). Early mutagenesis studies of TrpA suggested that the α subunit has a remarkable tolerance to amino-acid substitutions (for both solvent-exposed and buried residues) and this is consistent with the observation that TrpAs are relatively less conserved than TrpBs. The TrpA proteins show a pairwise sequence identity of only 25–33%, about half of that observed for TrpBs. From a large collection of *E. coli trpA* missense mutants in the N-terminal region only a few sites were found to be essential for function and/or structure (Creighton *et al.*, 1966 ▸; Lim *et al.*, 1991 ▸; Milton *et al.*, 1986 ▸).

The active site of subunit α, which in the *Sp*TrpA sequence comprises Glu52, Asp63, Tyr175, Gly213 and Gly234, catalyzes the retro-aldol cleavage of IGP using a push–pull general acid–base mechanism (Buller *et al.*, 2016 ▸). The active-site pocket is highly conserved and is located in the center of the TIM barrel (Supplementary Fig. S2; Juárez-Vázquez *et al.*, 2017 ▸). The barrel structure must be maintained for enzymatic activity, although sometimes it can be reconstituted from two peptides, as mentioned above. In the αββα complex the active site faces the β subunit and its activity is coordinated through interactions involving the β-subunit communication domain (COMM) and loops L2 and L6 from subunit α, with the latter adopting a closed state in the most active conformational arrangement of the heterotetramer.

About 50 years ago, Jackson and Yanofsky observed that wild-type *E. coli* TrpA (*Ec*TrpA) could be converted to a dimeric form *in vitro* by treating the monomer with urea and refolding. Dimers were also observed for some missense and nonsense mutants and peptide complementation (Jackson & Yanofsky, 1969*a* ▸,*b* ▸). It was hypothesized that such oligomerization might involve 3D domain swapping, but the very low yield (∼2%) has been a hindrance to further analysis of this structural phenomenon. The formation of TrpA dimers was also observed in site-specific mutants of *S. typhimurium* TrpA (*St*TrpA; Kim *et al.*, 2001 ▸). The wild-type enzyme produced a single, monomeric peak in size-exclusion chromatography (SEC); however, several double mutants (T24A/F139W, T24S/F139W and T24K/F139W) unexpectedly eluted not only as a monomer but also as a 52 kDa peak corresponding to an *St*TrpA dimer. CD measurements showed that the dimers and monomers had almost the same content of secondary structure, but tryptophan fluorescence suggested that the dimers had a larger exposed hydrophobic surface (Kim *et al.*, 2001 ▸). Mutation of residue 24 appeared to be essential for *St*TrpA dimer formation. However, this position is not well conserved among TrpA orthologs. Interestingly, dimerization-prone mutants also showed a twofold to eightfold reduced affinity for subunit β, but they retained the capability to bind subunit β and form functional αββα heterotetramers. To explain the dimer formation, 3D domain or loop swapping of an N- or C-terminal portion of *St*TrpA with a complementing molecule has been proposed (Kim *et al.*, 2001 ▸). Although TrpA dimers were not observed for the *E. coli* and *S. typhimurium* orthologs under normal conditions, mutagenesis and refolding results and sequence variability at position 24 of TrpA indicated that at least some variants could form dimers *in vivo*.

Here, for the first time, we provide structural proof that the biochemically observed dimerization of TrpA occurs *in vivo* via the long-postulated 3D domain swapping by presenting the crystal structure of the wild-type *Sp*TrpA ortholog. The canonical TIM-barrel fold of the protein is regenerated from two polypeptide chains exchanging the N-terminal fragment comprising the H0–S1–H1–S2 elements in a mutual fashion. The dimers are stable in solution, and in the crystal lattice they form a decameric assembly comprising two superposed pentameric rings. This work unequivocally demonstrates that proteins in the TIM-barrel family are also capable of exchanging portions of their structure to form stable dimers.

## 2. Materials and methods   

### 2.1. Gene cloning, protein expression and purification   

Gene cloning was performed according to Kim *et al.* (2011 ▸). Briefly, *S. pneumoniae* TIGR4 genomic DNA was used as a template for PCR amplification of the gene coding for the TrpA subunit (residues 1–258; *Sp*TrpA) of tryptophan synthase with the following primers in the reaction mixture: 5′-TACTTCCAATCCAATGCCATGCCTAAGACACTAACAGAAAAATTAAATGCTATTAAA-3′ and 5′-TTATCCACTTCCAATGTTATTTTTGGTAAGCTACTGCTTGTCTGATAAAATC-3′, as designed using an online tool (https://bioinformatics.anl.gov/targets/public_tools.aspx; Yoon *et al.*, 2002 ▸). The purified PCR products were treated with T4 DNA polymerase in the presence of dCTP (Eschenfeldt *et al.*, 2010 ▸) according to the vendor’s specification (New England Biolabs). The protruded DNA fragment of the *Sp*TrpA subunit was mixed with T4 DNA polymerase-treated vector pMCSG68 (PSI:Biology-Materials Repository) for ligation-independent cloning (Aslanidis & de Jong, 1990 ▸; Eschenfeldt *et al.*, 2009 ▸). *E. coli* BL21 Gold (DE3) cells were transformed with the amplified DNA/pMCSG68 mixture and were grown in the presence of ampicillin (150 µg ml^−1^) and kanamycin (25 µg ml^−1^). A single colony of each transformation was picked, grown and induced with isopropyl β-d-1-thiogalactopyranoside (IPTG). The construct was tested in small-scale expression and the sequence was verified.

To express selenomethionine (SeMet)-labeled *Sp*TrpA protein, starter cultures were grown overnight at 37°C and 200 rev min^−1^ in LB medium supplemented with 100 µg ml^−1^ ampicillin, 30 µg ml^−1^ kanamycin and 40 m*M* K_2_HPO_4_. The starter culture was used to inoculate 1 l enriched M9 medium for large-scale production of SeMet-labeled protein. The bacterial culture was grown at 37°C and 190 rev min^−1^ until it reached an OD_600_ of 1.0. After air-cooling to 4°C for 60 min, inhibitory amino acids (25 mg l^−1^ each of l-valine, l-isoleucine, l-leucine, l-lysine, l-threonine and l-phenyl­alanine) and 90 mg l^−1^ SeMet (Medicilon) were added. Protein expression was induced by 0.5 m*M* IPTG. The cells were incubated overnight at 18°C and harvested. Approximately 8 g of cell pellet was resuspended in lysis buffer [500 m*M* NaCl, 5%(*v*/*v*) glycerol, 50 m*M* HEPES pH 8.0, 20 m*M* imidazole, 10 m*M* β-mercaptoethanol] and stored at −80°C. *Sp*TrpA was purified using the procedure described previously (Kim *et al.*, 2004 ▸, 2011 ▸). The harvested cells were thawed and 1 mg ml^−1^ lysozyme was added. The mixture was kept on ice for 20 min with gentle shaking and was then sonicated. The lysate was clarified by centrifugation at 36 000*g* for 1 h and filtered through a 0.45 µm membrane. The clarified lysate was applied onto a 5 ml Ni HisTrap HP column (GE Healthcare Life Sciences) and the His_6_-tagged protein was released with elution buffer (500 m*M* NaCl, 5% glycerol, 50 m*M* HEPES pH 8.0, 250 m*M* imidazole, 10 m*M* β-mercaptoethanol). This was followed by buffer exchange on a Sephadex G-25 Fine XK 26/20 (GE Healthcare Life Sciences) desalting column equilibrated with buffer consisting of 20 m*M* Tris–HCl pH 7.5, 500 m*M* NaCl, 2 m*M* DTT. All of these steps were performed using an ÄKTAxpress chromatographic system (GE Healthcare Life Sciences). The fusion tag was removed by treatment with recombinant His_7_-tagged Tobacco etch virus (TEV) protease. Nickel-affinity chromatography was used to remove the His_6_ tag, uncut *Sp*TrpA protein and His_7_-tagged TEV protease (Blommel & Fox, 2007 ▸). The pure protein was concentrated in crystallization buffer *A* (20 m*M* HEPES pH 8.0, 250 m*M* NaCl, 2 m*M* DTT). The protein concentration was determined spectrophoto­metrically at 280 nm with a Nanodrop ND-1000 spectrophotometer (Thermo Fisher Scientific) using a calculated extinction coefficient of 8940 *M* ^−1^ cm^−1^.

### 2.2. Size-exclusion chromatography (SEC)   

A batch of *Sp*TrpA was subjected to an additional purification step via SEC on a Superdex 200 HiLoad 26/60 column (GE Healthcare Life Sciences) in crystallization buffer *A* directly following the second affinity-chromatography step. The sample was used for analytical experiments and follow-up crystallization trials. The column was calibrated with the following molecular-weight standards: ferritin (440 kDa), aldolase (158 kDa), conalbumin (75 kDa), carbonic anhydrase (29 kDa) and aprotinin (6.5 kDa).

### 2.3. Crystallization   

The SeMet *Sp*TrpA protein (46.6 mg ml^−1^) purified by affinity chromatography was screened for crystallization conditions using a sitting-drop vapor-diffusion setup at 4 and 24°C in CrystalQuick 96-well round-bottom plates (Greiner Bio-One). A 400 nl droplet of protein solution was mixed with 400 nl crystallization reagent and equilibrated over 135 µl crystallization reagent. The protein was screened with the MCSG1–3 screens (Anatrace). Nanopipetting was performed using a Mosquito nanolitre liquid-handling system (TTP Labtech). The plates were incubated in a RoboIncubator automated plate-storage system equipped with a Minstrel III automated crystal visualization system (Rigaku). The best crystals were obtained at 4°C in 31.7 m*M* sodium acetate, 5.56 m*M* Tris–HCl pH 8.5, 25% PEG 4K, 15% glycerol.

### 2.4. X-ray data collection and processing   

Single-wavelength anomalous X-ray diffraction (SAD) data were collected near the selenium edge on Structural Biology Center beamline 19-ID at the Advanced Photon Source (APS). 180 frames were collected with 1° oscillation and 2 s exposure per frame. Data processing was carried out in the *XDS* package (Kabsch, 2010 ▸). Friedel pairs were treated as different reflections, as a significant anomalous signal 〈*d*′′/sig〉 > 0.85 was present in the data up to 2.92 Å resolution. The unit-cell parameters and Bravais lattice were determined using the *COLSPOT* and *IDXREF* subroutines of *XDS*. The intensities were reduced to structure-factor amplitudes using the method of French & Wilson (1978 ▸) and were then converted to MTZ format using the *CCP*4 suite (Winn *et al.*, 2011 ▸). The processing parameters are summarized in Table 1 ▸.

### 2.5. Structure determination and refinement   

The crystal structure of *Sp*TrpA was solved at 2.54 Å resolution in space group *P*2_1_ by SAD phasing. The data collected near the selenium absorption peak energy (λ = 0.9793 Å) were fed into the *CRANK*2 pipeline (Skubák & Pannu, 2013 ▸; Winn *et al.*, 2011 ▸) running *SFTOOLS*, *SHELXC*/*D* (Sheldrick, 2015 ▸), *REFMAC*5 (Murshudov *et al.*, 2011 ▸), *MAPRO*, *SOLOMON* (Abrahams & Leslie, 1996 ▸), *MULTICOMB*, *Parrot* (Cowtan, 2010 ▸) and *Buccaneer* (Cowtan, 2006 ▸). The polypeptide chain of *Sp*TrpA contains three methionine residues in its sequence. *SHELXD* identified 42 heavy-atom sites (while 30 Se sites would be expected for ten protein chains per asymmetric unit). Only the first 26 sites were considered for phasing, as the subsequent sites had occupancies below 0.15. An initial atomic model of *Sp*TrpA was built using the *CCP*4 implementation of *Buccaneer* and was refined using *REFMAC*5, with additional manual corrections in *Coot* (Emsley *et al.*, 2010 ▸). Ultimately, ten protein chains were modeled in the asymmetric unit, assembled into five 3D domain-swapped dimers with the following labels: *AB*, *CD*, *EF*, *GH* and *IJ*.

The structure was refined with *REFMAC* v.5.8.0222 (Murshudov *et al.*, 2011 ▸) against maximum-likelihood targets, using the anomalous diffraction data from the SeMet derivative. Local NCS restraints and 80 TLS groups were used throughout the refinement. The standardized placement of the model in the unit cell was found with the *ACHESYM* server (Kowiel *et al.*, 2014 ▸). 1.2% of all reflections were selected for *R*_free_ testing. The standard protein stereochemical restraint library of Engh & Huber (1991 ▸) was used. *PyMOL* v.1.8 (Schrödinger) was used for structure visualizations and analysis.

## 3. Results and discussion   

### 3.1. Properties and structure analysis of *Sp*TrpA   

The reports by Jackson and Yanofsky on the observation of *Ec*TrpA dimers and mutational studies of *St*TrpA suggested that the TrpA protein might form stable dimers in some bacterial species (Jackson & Yanofsky, 1969*a* ▸,*b* ▸; Kim *et al.*, 2001 ▸). During our systematic studies of several TrpAB orthologs (Michalska *et al.*, 2019 ▸; Wellington *et al.*, 2017 ▸), the individual α and β subunits of *S. pneumoniae* TrpAB were expressed in *E. coli*, as well as being coexpressed. Interestingly, when the α subunit alone was produced and purified, it separated into two forms on SEC, an ∼30 kDa monomer and an ∼68 kDa dimer, with the monomer:dimer ratio being dependent on the sample batch (Supplementary Fig. S3*a*). The monomer and dimer fractions could also be separated on native PAGE, with one protein band observed for the monomer and two protein bands (monomer and dimer) observed for the dimer fraction (Supplementary Fig. S3*b*). This suggested that the homodimers generated during the expression of the α subunit in *E. coli* represent an abundant form. Purified monomer equilibrated into a monomer–dimer mixture and vice versa: purified dimer equilibrated into a similar mixture. Incubation of the monomer or the dimer with 1 *M* urea resulted in the formation of monomers only (Supplementary Fig. S3*b*).

The dimeric fraction of the monomer–dimer mixture was crystallized in the monoclinic space group *P*2_1_ with ten chains of *Sp*TrpA in the asymmetric unit. The structure of *Sp*TrpA was solved by single-wavelength anomalous X-ray diffraction (SAD) phasing and refined to 2.54 Å resolution (Table 1 ▸). A nearly complete polypeptide could be traced for all chains, with the exception of residues in the Ile179–Ala191 range, which showed disorder. Only polypeptide chain *C* could be fully modeled, albeit with high *B* factors. In chain *B*, a fragment of the N-terminal affinity tag (SNA–) could also be traced.

### 3.2. 3D domain swapping in the homodimer   

**Figure 1:**
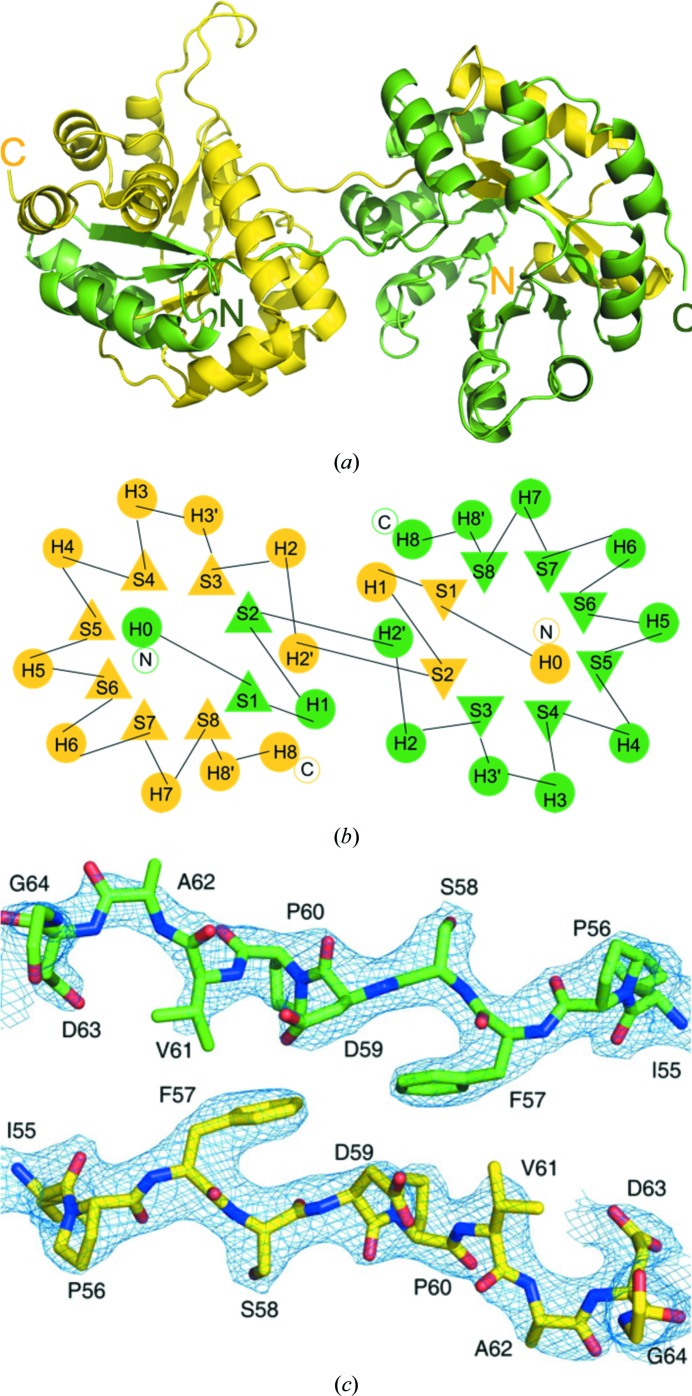
The *Sp*TrpA 3D domain-swapped dimer. (*a*) The 3D domain swapping of protein chains *C* (yellow) and *D* (green). (*b*) Topology of the secondary-structure elements viewed down the axis of the 3D domain-swapped TIM barrel of *Sp*TrpA. Helices are marked as circles and labeled H0–H8; β-­strands are marked as triangles and labeled S1–S8. Residue ranges for the α-helices and β-strands are marked in Supplementary Fig. S1. (*c*) The domain-swapping hinge (residues Ile55–Gly64) of chain *C* (yellow) and chain *D* (green), shown as 2*F*_o_ − *F*_c_ electron density contoured at 1.2σ.

The asymmetric unit comprises five α_2_ dimers of *Sp*TrpA formed via 3D domain swapping (Figs. 1 ▸ and 2 ▸). As in other TIM-barrel structures, the chain topology of *Sp*TrpA consists of eight parallel β-strands (labeled S1–S8) forming the internal β-barrel, which are separated and flanked on the outside of the barrel by helices H1–H8 from the β–α–β motifs (Figs. 1 ▸ *b* and 2 ▸ *b*). Helix H2′ is not part of the barrel but is located on top of it and leads directly to the domain-swapping hinge (Supplementary Fig. S4). The β–α–β motif involving strands S3 and S4 is formed with the participation of two helices (H3′–H3). The progression of the β–α–β motifs is preceded by the N-terminal helix H0 located at the bottom of the TIM barrel and is followed by a tandem of helices, H8′–H8, of which helix H8′ is again located at the top of the barrel. In addition, *Sp*TrpA has a small insertion in loop L1 that appears to render the 3D domain-swapped dimers more stable (Supplementary Fig. S5). This feature is only present in TrpA sequences from *Streptococcus*, *Lactoccocus* and *Floricoccus* species.

**Figure 2:**
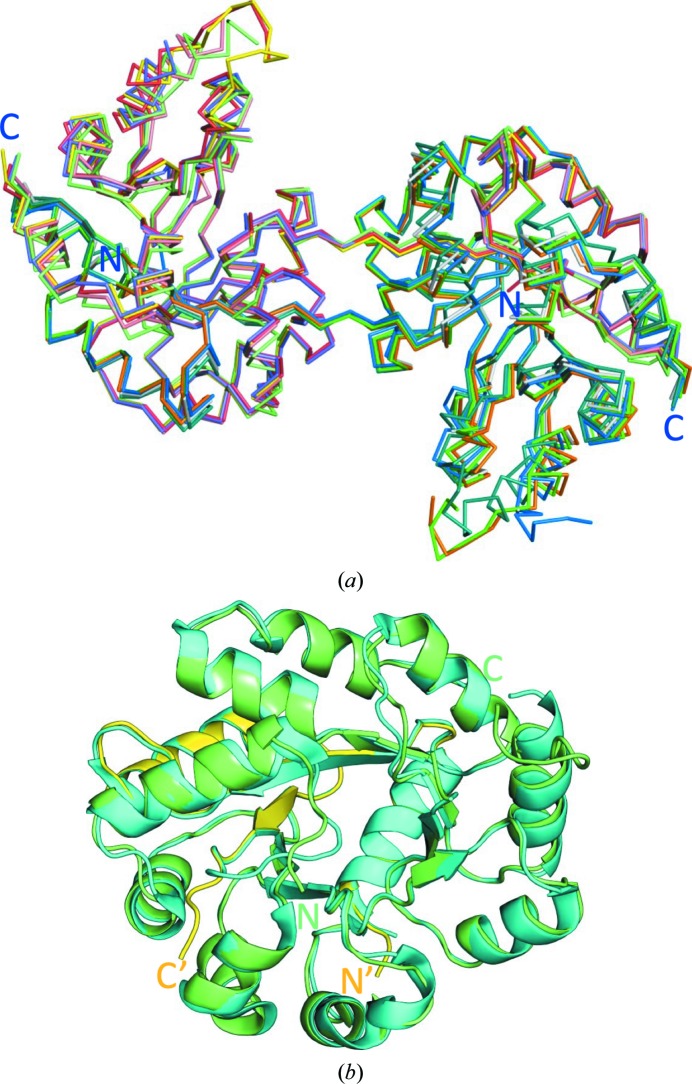
Comparison of *Sp*TrpA dimers and the TIM-barrel fold. (*a*) C^α^ superposition of the five dimers: *A*/*B*, red/blue; *C*/*D*, yellow/green; *E*/*F*, salmon/gray; *G*/*H*, violet/orange; *I*/*J*, light green/teal. The superposition was calculated using the *align* procedure in *PyMOL*. (*b*) Superposition of the TIM barrel of *Sp*TrpA formed from chains *C* (residues 1–59, yellow) and *D* (60–258, green) with the model of the *Sp*TrpA subunit (cyan) from the structure of *Sp*TrpAB (PDB entry 5kin; r.m.s.d. of 0.45 Å on C^α^ superposition).

The five dimers in the asymmetric unit have a similar structure (Fig. 2 ▸), as illustrated by the r.m.s.d. values for pairwise superpositions of their C^α^ atoms, which vary from 0.43 Å (for superposition of *AB* with *CD*) to 1.61 Å (*GH* with *IJ*) (Supplementary Table S1). The molecules assemble into a doughnut-like decamer or a pentamer of dimers (*AB*, *CD*, *EF*, *GH* and *IJ*; Fig. 4) as described below.

### 3.3. The active site of the *Sp*TrpA dimer and comparison with similar structures   

**Figure 3:**
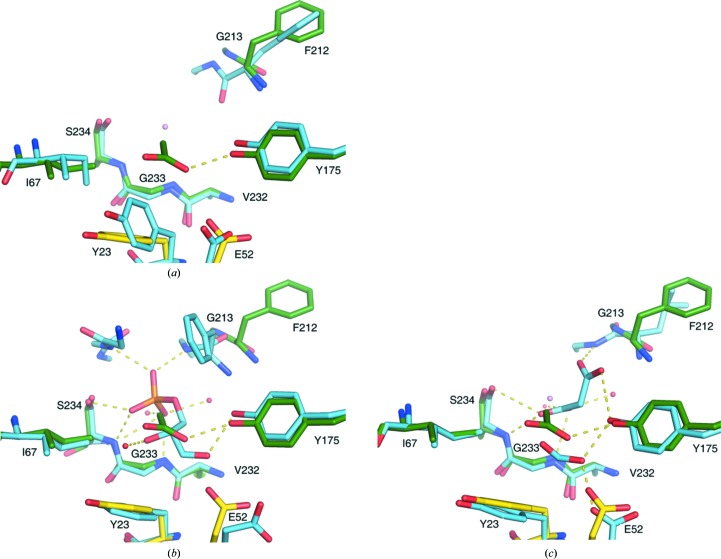
Details of the *Sp*TrpA active site. The active site of *Sp*TrpA, chain *C* (yellow) and chain *D* (green), including the acetate ion (ACY; green) superposed with (*a*) chain *A* of the *Sp*TrpAB structure (PDB entry 5kin; blue), (*b*) chain *A* of the *St*TrpA structure (PDB entry 1wbj; blue) including the *sn*-glycerol-3-phosphate ligand (G3P; blue), with water molecules from the *St*TrpA structure shown as red spheres, and (*c*) chain *A* of the *Mt*TrpAB structure (PDB entry 5tcf; blue) including the formic acid and malonate ligands (FMT and MLI; blue), with water molecules from the *Mt*TrpAB structure shown as red spheres. Selected hydrogen bonds are marked as yellow dashes. A water molecule from the present *Sp*TrpA structure is shown as a pink sphere.

One half of the dimer (the TIM-barrel folding unit), which consists of residues Mse1–Asp59 from one chain (for example *A*) and residues Pro60–Lys258 from the other chain (*B*) of the dimer, has a very similar fold to the α subunit from the αββα assembly of the TrpAB structure (PDB entry 5kin; Michalska *et al.*, 2019 ▸) from the same organism (*Sp*TrpAB) (Fig. 2 ▸ *b*). The r.m.s.d. for C^α^ superposition of the TIM barrel of *Sp*TrpA formed by one half of the *CD* dimer (*C*, Mse1–Asp59; *D*, Pro60–Lys258) with the α-subunit chain *A* of the *Sp*TrpAB complex is 0.45 Å. As is usually the case, domain swapping does not change the overall fold of the folding unit (the TIM barrel in this case). The only visible change is in the conformation of the short linker (Ile55–Asp64) connecting the swapped domains. The active site in each TIM barrel is restored and two α chains now contribute residues to each active site in the dimer (Fig. 3 ▸). In fact, the positions of the side chains of the conserved active-site residues (Glu52, Asp63, Tyr175, Gly213, Gly233 and Ser234) in the individual α subunits of the α_2_ and αβ dimers are very similar. In our *Sp*TrpA structure, as well as in the *Sp*TrpAB structure, Phe212 is in the open conformation (Fig. 3 ▸; Michalska *et al.*, 2019 ▸). It appears that upon ligand binding the Phe212 residue could adopt a closed conformation (Fig. 3 ▸ *b*). Additionally, in the *St*TrpAB structure (PDB entry 1wbj; Kulik *et al.*, 2005 ▸) the *sn*-glycerol-3-phosphate ligand is bound by Gly184. However, in the present *Sp*TrpA model this part of the structure is in open conformation. Since there is no corresponding ligand in the active-site pocket in our structure, we cannot confirm the exact role of the disordered flexible loop L6 (residues ∼180–190) in ligand binding. We can only speculate that it could either move towards the active site and increase the affinity for the phosphate moiety of a ligand, or it could remain disordered when the ligand is present in the active site, leaving the active site fully open. The active site of *Sp*TrpA is also very similar to that of the *Mycobacterium tuberculosis* TrpAB (*Mt*TrpAB) structure (PDB entry 5tcf; Wellington *et al.*, 2017 ▸), including the location of the acetate ion present in the *Sp*TrpA structure, which occupies the same position as a formate ion in the *Mt*TrpAB model (Fig. 3 ▸). Based on data available for *S. typhimurium* TrpA dimers and the overall conservation of the active site, it seems very likely that the *Sp*TrpA dimers should be able to convert IGP to indole.

### 3.4. The first case of a TIM barrel formed with 3D domain swapping   

3D domain swapping is a mechanism of protein oligomerization in which two (or more) protein chains mutually exchange structural elements in a circular or linear runaway fashion. In the simplest case, two monomers exchange a fragment of their structures. This fragment may consist of a single secondary-structure element, for example an α-helix, or a globular domain. If the exchange is reciprocal between two protomers, symmetric dimers are formed. Domain swapping was first proposed for, and was later observed in, RNase A (Crestfield *et al.*, 1962 ▸; Liu *et al.*, 1998 ▸). The first structural evidence of 3D domain swapping was reported by Bennett, Choe and Eisenberg (Bennett *et al.*, 1994 ▸) for dimeric diphtheria toxin and this phenomenon has since been described in a number of reviews (Jaskólski, 2001 ▸, 2013 ▸). Since its discovery, 3D domain swapping has been reported for a large number of proteins (over 500 structures are available in the PDB) with diverse folds but, to the best of our knowledge, never for an intrinsic portion of a TIM barrel. There are nearly 2000 TIM-barrel structures in the PDB, representing over 300 unique sequence families (<30% identity), including computationally designed proteins. This is a stable fold and some TIM barrels are highly ordered; for example, crystals of human aldose reductase with this fold diffracted X-rays to 0.62 Å resolution (Howard *et al.*, 2004 ▸). There are cases reported in the literature and in the PDB of TIM-barrel proteins undergoing 3D domain swapping of an accessory (usually C-terminal) appending structure (usually an α-helix), but domain swapping in these cases does not disrupt the integrity of the compact TIM-barrel fold (http://caps.ncbs.res.in/3dswap/; Shameer *et al.*, 2011 ▸). For instance, a C-terminal helix swap (the first such case to be reported) leads to the dimerization of phosphoenolpyruvate mutase (PDB entry 1pym; Huang *et al.*, 1999 ▸) or produces cyclic pentamers of fructose-6-phosphate aldolase (FSA; PDB entry 1l6w; Thorell *et al.*, 2002 ▸). The TIM-barrel core itself, however, does not seem to be very amenable to structural 3D domain swapping. It is thus remarkable to find in the present structure of *Sp*TrpA that this kind of structural rearrangement is indeed possible. Moreover, many of the previous examples with accidental TIM-barrel involvement in oligomerization are not bona fide 3D domain-swapping cases because the monomeric form does not exist. In the domain-swapping of *Sp*TrpA, the N-terminal structural elements (N)–H0–S1–H1–S2 (with the sequence Mse1–Asp59) are contributed by the complementary subunit (Fig. 1 ▸, Supplementary Fig. S4). Despite the domain swap, the TIM-barrel topology is fully preserved and is identical to that found in monomeric *Sp*TrpA (Fig. 2 ▸ *b*, Supplementary Fig. S4), satisfying the strictest definition of 3D domain swapping. The linker or hinge region comprises the mostly hydrophobic Ile55–Gly64 decapeptide. In the quaternary structure of *Sp*TrpA, the two linker chains form an antiparallel structure, which is the new open interface of the dimer (Fig. 1 ▸ *c*, Supplementary Fig. S4; Bennett *et al.*, 1995 ▸). The excellent electron density of the linker region Ile55–Gly64 (Fig. 1 ▸ *c*) leaves no doubt that 3D domain swapping has occurred. Surprisingly, at variance with other similar cases, for example the 3D domain-swapped dimers of human cystatin C (Janowski *et al.*, 2001 ▸), these antiparallel linker chains are not connected by hydrogen bonds and do not form a β-sheet structure, even though they assume an extended conformation. Thus, the open interface seems to be stabilized mostly by hydrophobic interactions. The tight closed interface, which is part of the compact TIM-barrel fold, is identical to that in the monomeric protein. The Ramachandran angles of the linker peptides are similar in all eight copies of the *Sp*TrpA chain (Supplementary Table S2) and in general correspond to an extended conformation, which is however quite distant from the typical β structure (φ ≃ −120°, ψ ≃ +120°). The two C-terminal residues of the linkers are no longer classified in the Ramachandran β region. Asp63 with its negative ψ angle is found in the α conformation. Gly64 with its positive φ angle lies in a disallowed Ramachandran region that is however possible for glycine residues.

The TrpA chains in the domain-swapped dimers are (pseudo)twofold-symmetric, as illustrated by the ∼180° rotation required to superpose the subunits, which is practically the same for all of the dimers. The local dyads pass between the two linker chains of the open interface, close to the Asp59–Pro60 peptides.

The hinge-loop region involved in the domain swapping is the only part that adopts a different conformation in the monomeric and domain-swapped forms (Supplementary Fig. S4). Frequently, proline residues are found in the hinge region, suggesting their possible importance, as prolines can create strain and thereby favor domain swapping (Huang *et al.*, 2018 ▸). It has been shown that replacement of the first proline by alanine in the hinge region of the p13suc1 protein stabilizes the monomer, whereas the same substitution of the second proline stabilizes the dimer (Rousseau *et al.*, 2001 ▸). In *Sp*TrpA there are two proline residues in the hinge region (Pro56 and Pro60) that are quite conserved among TrpA homologs (Supplementary Fig. S5). Pro60 is located in the Ramachandran α region. Interestingly, it has been shown that a *cis*-prolyl peptide-bond isomerization at this position dominates the folding of the α subunit of tryptophan synthase (Wu & Matthews, 2002 ▸).

It has long been recognized that hinge elements that are too short, and also those that are too long, may be an obstacle to productive 3D domain swapping. This might be the reason why the robust TIM-barrel fold is resistant to oligomerization via 3D domain swapping; the tight loops of the β–α–β crossovers may be too short to connect two barrels without steric clashes, while the extended loops at the business side of the barrel may be too long. The presence of the surface-protrusion helix H2′ in the N-terminal part of the TrpA fold is a topological novelty that makes the S2–H2′ loop a suitable hinge for 3D domain swapping (Supplementary Figs. S5 and S6). The S2–H2′ linker is quite long, and in the structure of the *Sp*TrpA monomer (PDB entry 5kin) has a manifold structure with three sharp kinks: first moving away from helix H2, then turning ‘down’ and finally turning again to join helix H2′ (Supplementary Fig. S6). In its domain-swapped (open) conformation the linker runs straight ahead to reach helix H2′ in the complementary folding unit directly. As illustrated in Supplementary Fig. S6, in the hypothetical situation of an absent helix H2′ the switch from β S2 to helix H2 would require the two linkers to cross, a maneuver preventing 3D domain swapping (Supplementary Fig. S6). A comparison of a ‘simple’ 3D domain-swapping event with a more convoluted event (as in TrpA), in which the hinge has to undergo a significant conformational change in order to reach the complementary folding unit and avoid clashes with its own copy, is illustrated in Supplementary Fig. S6.

It is interesting to note that a similar protruding helix (H8′) exists in the C-terminal part of the *Sp*TrpA TIM barrel. This suggests the interesting possibility of 3D domain swapping with the utilization of two swapped domains. Such a phenomenon, although rare, is known to be conducive to linear polymerization via 3D domain swapping (Liu *et al.*, 1998 ▸) and thus to the formation of fibrillar protein aggregates, which typically show up on SDS–PAGE electrophoretograms as high-mass smears (Wahlbom *et al.*, 2007 ▸). One may speculate that the high-molecular-mass peak shown by SEC in Supplementary Fig. S3 could reflect such linear polymers or indeed oligomers formed by runaway propagation of the N-terminal 3D domain swap described in this work.

How can we explain the propensity of *Sp*TrpA for 3D domain swapping? It appears that the N- and C-terminal regions of TrpA have rather different properties. Folding studies of *St*TrpA indicated that the very N-terminal region of the protein corresponding to the H0–S1–H1–S2 secondary-structure elements folds rapidly and is tightly packed. In contrast, the C-terminus appears to behave as a molten globule whose folding is strongly coupled to that of the H0–S1–H1–S2 domain. In asymmetric deletion experiments, when the N-terminal fragment terminates at residue 96, the C-terminal fragments tend to start before residue 73. Similarly, when the C-terminal fragment starts at residues 57 or 73, all complementary N-terminal fragments terminate after residue 68 (Hiraga *et al.*, 2004 ▸). This region corresponds to the H0–S1–H1–S2 structural unit and is also related to the unit boundary at position 73 (H2′ helix; Hiraga *et al.*, 2004 ▸). Perhaps a tightly packed N-terminal region when combined with a longer loop L1 (or some mutations) is conducive to 3D domain swapping. The presence of helix H2′ also makes 3D domain swapping feasible. Therefore, under certain conditions the N-terminal fragment can connect to the C-terminal fragment at this boundary in the TIM-barrel structure. Statistical analysis of the N- and C-terminal fragment complementations that can restore α-subunit activity showed that accumulated probability is lowest for N-terminal fragments of less than 57 residues (Wu *et al.*, 2005 ▸, 2007 ▸). This is also supported by TrpA mutants in which the catalytic residues are mutated (E49G, D60G and D60N), which do not support growth *in vivo*. Moreover, some enzymatically defective mutants of the α subunit are also defective in their ability to activate the β subunit. The mutation of the conserved, noncatalytic Asp56 to Gly in *Ec*TrpA causes a nearly total loss of activity in both the α and αβ reactions. Nevertheless, with the exception of α-subunit mutants altered at the catalytic site residues (Glu49 and Asp60 in *Ec*TrpA), other mutants can support growth, including substitutions at positions 18, 48 and 66. These results are also consistent with earlier *Ec*TrpA peptide and fragment complementation and circular-permutation results (Hiraga *et al.*, 2004 ▸).

### 3.5. The decameric quaternary structure of *Sp*TrpA   

**Figure 4:**
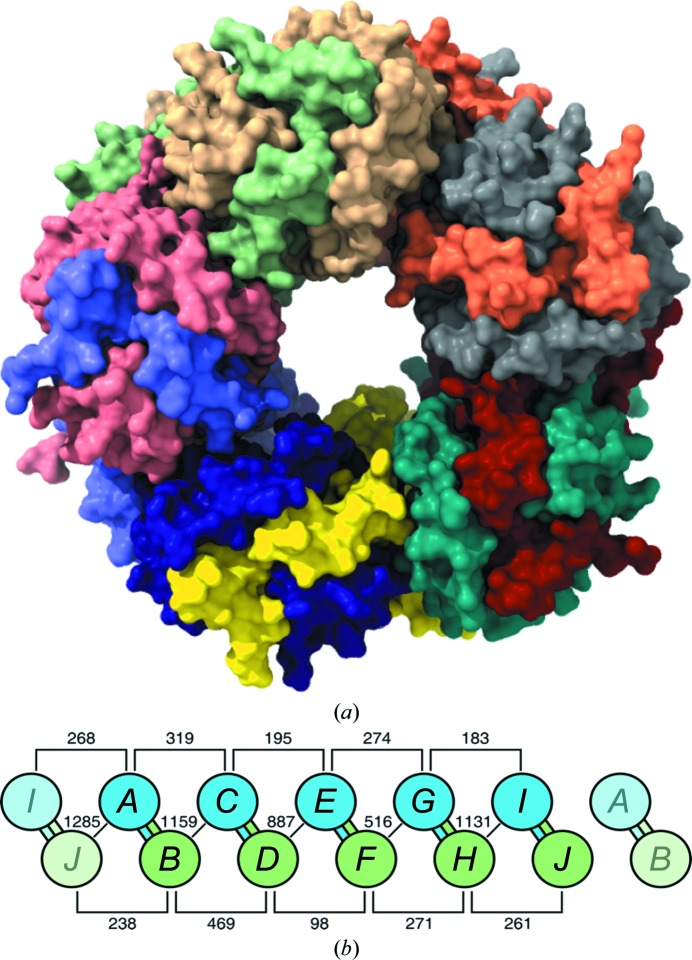
Structure of the *Sp*TrpA decamer. (*a*) Contents of the asymmetric unit viewed down the fivefold axis of the decamer. Chain colors are as in Fig. 2 ▸(*a*). (*b*) The domain-swapped dimers are formed by the following pairings: *AB*, *CD*, *EF*, *GH* and *IJ*. The decamer comprises two pentameric rings: –­*A*–*C*–*E*–*G*–*I*– (blue) and –*B*–*D*–*F*–*H*–*J*– (green). To illustrate the circular connectivity, the unique subunit sequences (for example *A*–*C*–*E*–*G*–*I*–) are flanked by their circular neighbors (for example *I* and *A*) shown in dim colors. Chain surface interface (buried) areas are given in Å^2^, as calculated by the *PISA* server (Krissinel & Henrick, 2007 ▸). The mean interface area between chains forming the α_2_ domain-swapped dimers (*AB*, *CD*, *EF*, *GH* and* IJ*) is ∼5271 Å^2^. The variabilities of the buried surface areas in the same group (for example *I*/*A*, *J*/*B*, *A*/*C* *etc.*) reflect to a certain degree the variable incompleteness (missing fragments) of the models.

The asymmetric part of the unit cell comprises a doughnut-like decamer or pentamer of dimers (*AB*, *CD*, *EF*, *GH* and *IJ*; Fig. 4 ▸). Chains *A*, *C*, *E*, *G* and *I* create a circular pentamer (with a subunit rotation of 72°) at the top of this aggregate, whereas the pentamer created by chains *B*, *D*, *F*, *H* and *J* forms the bottom ring, with an ∼36° rotation relative to the top pentamer. It is possible to superpose the two rings with a 180° rotation around a twofold axis (*i.e.* one of the dyads of the domain-swapped dimers) perpendicular to the main fivefold axis of the doughnut (for example, after such a rotation the subunit matching could be *A*–*B*, *C*–*J*, *E*–*H*, *G*–*F* and *I*–*D*). According to the *PISA* server (Krissinel & Henrick, 2007 ▸), the mean interface area between the subunits forming the 3D domain-swapped α_2_ dimers is ∼5271 Å^2^, which is ∼31.5% of the mean solvent-accessible surface area of an isolated chain in the extended (*i.e.* domain-swap-compatible) conformation. The interface areas between pairs of adjacent chains from the two pentamers not involved in 3D domain swapping vary from 516 Å^2^ (pair *F*–*G*) to 1285 Å^2^ (pair *A*–*J*). The interface area between consecutive chains within one pentamer is smaller and varies from 98 Å^2^ (*D*–*F*) to 469 Å^2^ (*B*–*D*) (Fig. 4 ▸ *b*).

It is interesting to note that FSA, which is a TIM barrel with a C-terminal helix swap, also forms a decameric assembly composed of a stack of two pentameric rings (PDB entry 1l6w). In this case, however, the 3D domain-swapping pattern is circular, connecting the protomers of the pentameric rings, while in *Sp*TrpA 3D domain swapping links subunits from both rings.

The main axis of the decameric doughnut is inclined at ∼43.5° to the crystallographic 2_1_ axis. This leads to a crystal packing in which the adjacent doughnut rings are inclined to each other by about 90°. The local pseudo-twofold axes (five copies) around the decameric structure are inclined at ∼90° to the principal fivefold axis of the decamer. Despite these interesting geometrical features, we believe that in the light of the small decamer-specific interface areas (as compared with the surface buried on 3D domain-swapping dimerization), the observed *Sp*TrpA decamer is likely to be an artifact of crystallization.

Are the dimerization properties of TrpA of functional significance, or is this just a transitional byproduct of some mutation(s) with little impact on l-tryptophan biosynthesis? Analysis of TrpA genes suggests that only a fraction of TrpA proteins may have a strong propensity to form homodimers. If the presence of the L1 insertion is a reliable indicator, 3D domain swapping may be prominent in TrpA from Gram-positive cocci strains, although mutagenesis experiments with *St*TrpA showed that dimers can also form in the absence of L1 loop insertion and that domain-swapped dimers should be more stable because of a larger interaction surface.

## 4. Conclusions   

We have determined the crystal structure of *S. pneumoniae* TrpA, the α subunit of tryptophan synthase, a key enzyme in tryptophan biosynthesis, and have discovered that it exists in solution and in the crystal as a very unusual dimer formed by swapping the N-terminal H0–S1–H1–S2 portion of the TIM-barrel core. Previous folding studies (Wintrode *et al.*, 2005 ▸) have shown that this region of TrpA folds rapidly and is tightly packed, providing a convincing rationale for exchanging this stable subdomain. For the first time, we provide structural evidence that the dimerization of TrpA, which was originally discovered by Jackson and Yanofsky, occurs via a long-postulated 3D domain swapping. Moreover, this is the first structural proof of bona fide 3D domain swapping in the core of a TIM barrel, which is one of the most abundant, versatile, robust and adaptable protein folds in nature and the subject of countless structural studies. The canonical TIM-barrel fold of the protein is regenerated from two polypeptide chains exchanging the N-terminal region. The hinge linker corresponds to loop L2 joining strand S2 to helix H2′. The structural elements S2 and L2 carry the key catalytic residues Glu52 and Asp63. As the S2 element is part of the swapped domain, the architecture of the catalytic apparatus in the α_2_ dimer is recreated from two protein chains. In solution the α_2_ dimer can be separated from the monomer by SEC, but it is in equilibrium with the monomer, which is then available for interaction with the β subunit to form the functional tryptophan synthase αββα complex. In the crystal, the 3D domain-swapped α_2_ dimers assemble into a decamer comprising two superposed pentameric rings. Our work unequivocally demonstrates that TIM-barrel proteins are also capable of exchanging portions of their core structure to form stable dimers and perhaps even higher-order oligomers. The dimerization may increase the enzyme stability and serve as a reservoir of α subunits and as an alternative source of indole from IGP for metabolic pathways that have still to be discovered.

## Supplementary Material

PDB reference: domain-swapped TrpA, 6qky

Supplementary Tables and Figures. DOI: 10.1107/S2059798320000212/wa5122sup1.pdf

## Figures and Tables

**Table 1:** Data-processing and refinement statistics Values in parentheses are for the highest resolution shell.

Data processing	
Beamline	19-ID, APS
Wavelength (Å)	0.9793
Resolution range (Å)	48.99–2.54 (2.68–2.54)
Space group	*P*2_1_
Unit-cell parameters (Å, °)	*a* = 104.81, *b* = 138.62, *c* = 107.25, β = 117.21
Unique reflections	171460 (24979)
Multiplicity	1.93 (1.95)
Completeness (%)	97.1 (98.4)
〈*I*〉/〈σ(*I*)〉	9.53 (1.26)
CC_1/2_ (%)	99.7 (40.8)
Wilson *B* factor (Å^2^)	73.8
*R*_merge_ †	0.056 (0.968)
Refinement	
Resolution range (Å)	48.99–2.54
No. of reflections (work/test set)	169369/2091
*R*_work_/*R*_free_ ‡	0.1930/0.2344
Average *B* factors (Å^2^)	
Overall	79.3
Macromolecules	79.6
Buffer molecules (ACY, GOL, PEG)	88.5
Water molecules	57.4
No. of atoms	
Total	19389
Macromolecules	18978
Buffer molecules (ACY, GOL, PEG)	72
Water molecules	339
R.m.s.d., bonds (Å)	0.016
R.m.s.d., angles (°)	1.53
Ramachandran plot§	
Favored (%)	97.8
Outliers (%)	0
Clashscore§	4
PDB code	6qky

† *R*_merge_ = 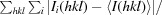 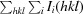, where *I*_*i*_(*hkl*) is the intensity of observation *i* of reflection *hkl*.

‡ *R*_work_ = 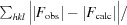 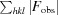 for all work reflections, where *F*_obs_ and *F*_calc_ are the observed and calculated structure factors, respectively. *R*_free_ is calculated analogously for the test reflections, which were randomly selected and were excluded from the refinement.

§ As defined by *MolProbity* (Chen *et al.*, 2010 ▸).
