# Supplementary material for: 3D domain swapping in the TIM barrel of the α subunit of Streptococcus pneumoniae tryptophan synthase
Source: Acta Crystallogr D Struct Biol. 2020 Jan 31;76(Pt 2):166–75. doi: 10.1107/S2059798320000212 (PMC7008512; doi:10.1107/S2059798320000212)
Supplement: Supplementary file 1 [file d-76-00166-sup1.pdf]

STRUCTURAL
BIOLOGY

Volume 76 (2020)

Supporting information for article:

3D domain swapping in the TIM barrel of the α subunit of *Streptococcus pneumoniae* tryptophan synthase

Karolina Michalska, Marcin Kowiel, Lance Bigelow, Michael Endres, Miroslaw Gilski, Mariusz Jaskolski and Andrzej Joachimiak

Table S1 RMSD (Å) for pairwise Cα superpositions of the dimers (with the numbers of superposed atoms in parentheses). The superpositions were calculated using the *align* procedure of PYMOL.

Dimer	CD	EF	GH	IJ
**AB**	0.43 (444)	0.83 (431)	0.80 (461)	1.55 (485)
**CD**		0.74 (437)	0.62 (487)	1.52 (482)
**EF**			0.56 (411)	1.09 (463)
**GH**				1.61 (462)

Table S2 Ramachandran φ/ψ angles (°) of the hinge region Ile55-G64 of the A-J subunits of the 3D domain-swapped dimers.

φ /ψ	I55	P56	F57	S58	D59	P60	V61	A62	D63	G64
**A**	-104/127	-68/146	-92/122	-113/137	-78/152	-91/0	-63/124	-134/27	-84/-17	99/166
**B**	-103/127	-68/145	-90/123	-106/133	-72/136	-94/-1	-71/125	-133/26	-82/-17	98/166
**C**	-104/127	-69/147	-90/124	-110/138	-75/151	-92/0	-66/125	-134/27	-84/-17	99/167
**D**	-103/127	-68/146	-91/122	-107/131	-72/136	-93/-1	-71/126	-132/26	-81/-20	99/163
**E**	-102/128	-68/145	-92/123	-107/132	-75/138	-91/-1	-69/126	-133/27	-85/-15	97/167
**F**	-103/128	-69/145	-92/123	-108/134	-70/137	-90/0	-65/126	-133/26	-85/-15	99/170
**G**	-103/127	-68/146	-91/120	-108/132	-79/146	-92/-1	-68/125	-138/31	-83/-16	100/166
**H**	-104/127	-69/146	-91/122	-111/135	-77/151	-92/1	-65/125	-132/26	-82/-18	99/166
**I**	-102/127	-66/143	-96/123	-107/130	-72/138	-95/-1	-67/124	-133/27	-85/-15	99/170
**J**	-103/127	-66/143	-96/122	-108/130	-79/147	-91/-3	-69/126	-132/25	-85/-16	97/167

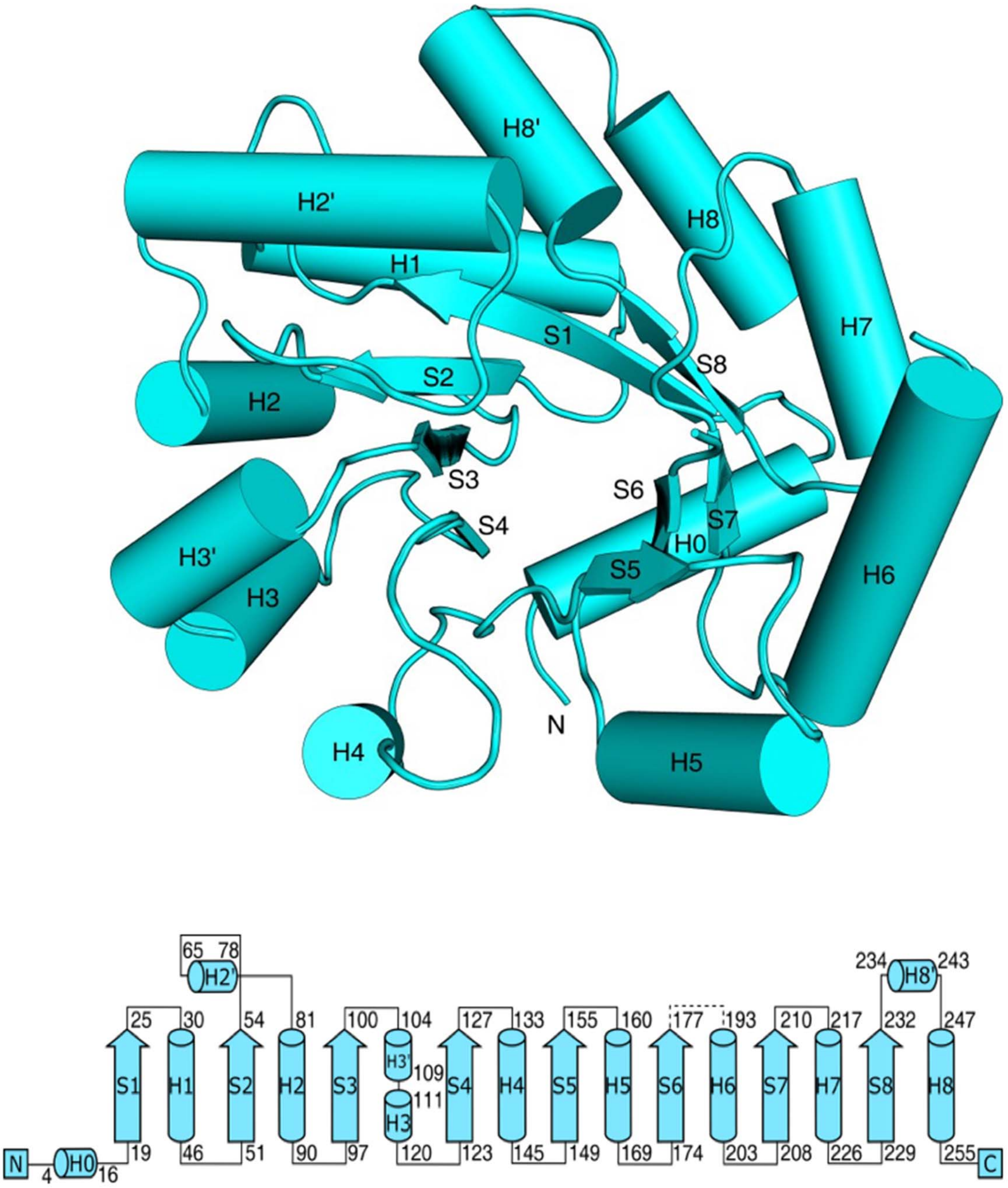

Figure S1 A view of the subunit α TIM barrel from the *Sp*TrpAB PDB structure (PDB id: 5KIN) (chain A) down the barrel axis (top) and the topology of the secondary structure elements (bottom). Helices are marked as cylinders and labeled H0-H8, β-strands are marked as arrows and labeled S1-S8. Residue ranges for the α-helices and β-strands are marked next to the cylinders and arrows. The disordered part of the structure is marked with dash line.

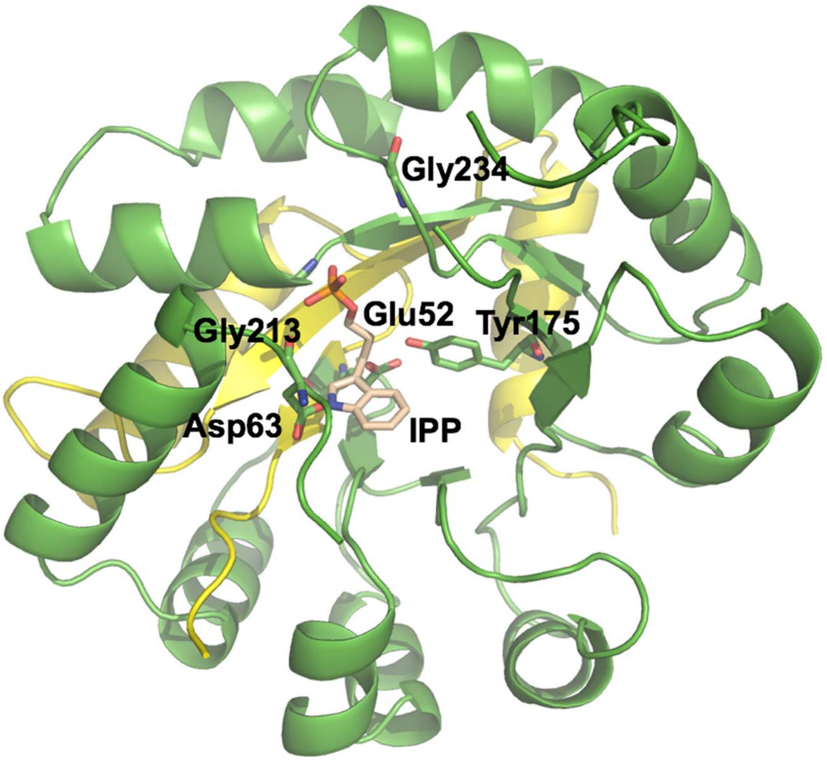

Figure S2 TrpA active site with key catalytic residues. The architecture of the catalytic apparatus in α_2 is regenerated from the two protein chains, C (residues 1-59, yellow) and D (residues 60-258, green). Indol-3-propanol phosphate (teal) from the *St*TrpAB structure (PDB id: 1QOP) (Juarez-Vazquez *et al.*) marks the position of the IGP substrate.

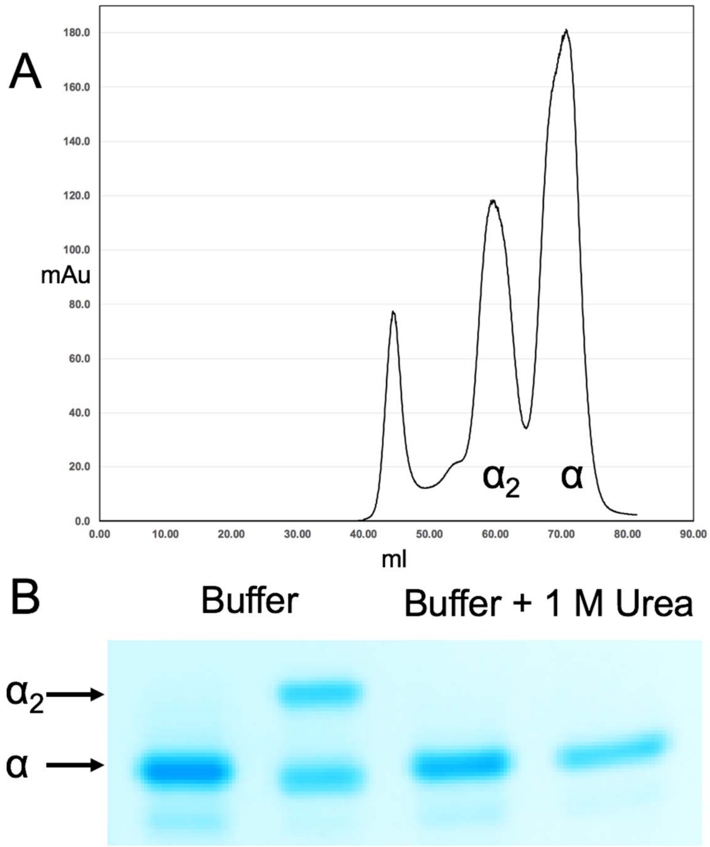

Figure S3 Separation of *Sp*TrpA on SEC and native PAGE. (A) SEC on Superdex 200 HiLoad 26/60 column in 250 mM NaCl, 20 mM HEPES pH 8.0, 2 mM DTT as described in Materials and methods. (B) Native PAGE electrophoretogram showing separation of *Sp*TrpA monomer (α) and *Sp*TrpA dimer (α_2). The dimer fraction after purification always showed some monomer present. After treatment with 1 M urea all dimers are converted to monomers. The buffer was 50 mM NaCl, 20 mM HEPES pH 8.0 and 2 mM DTT.

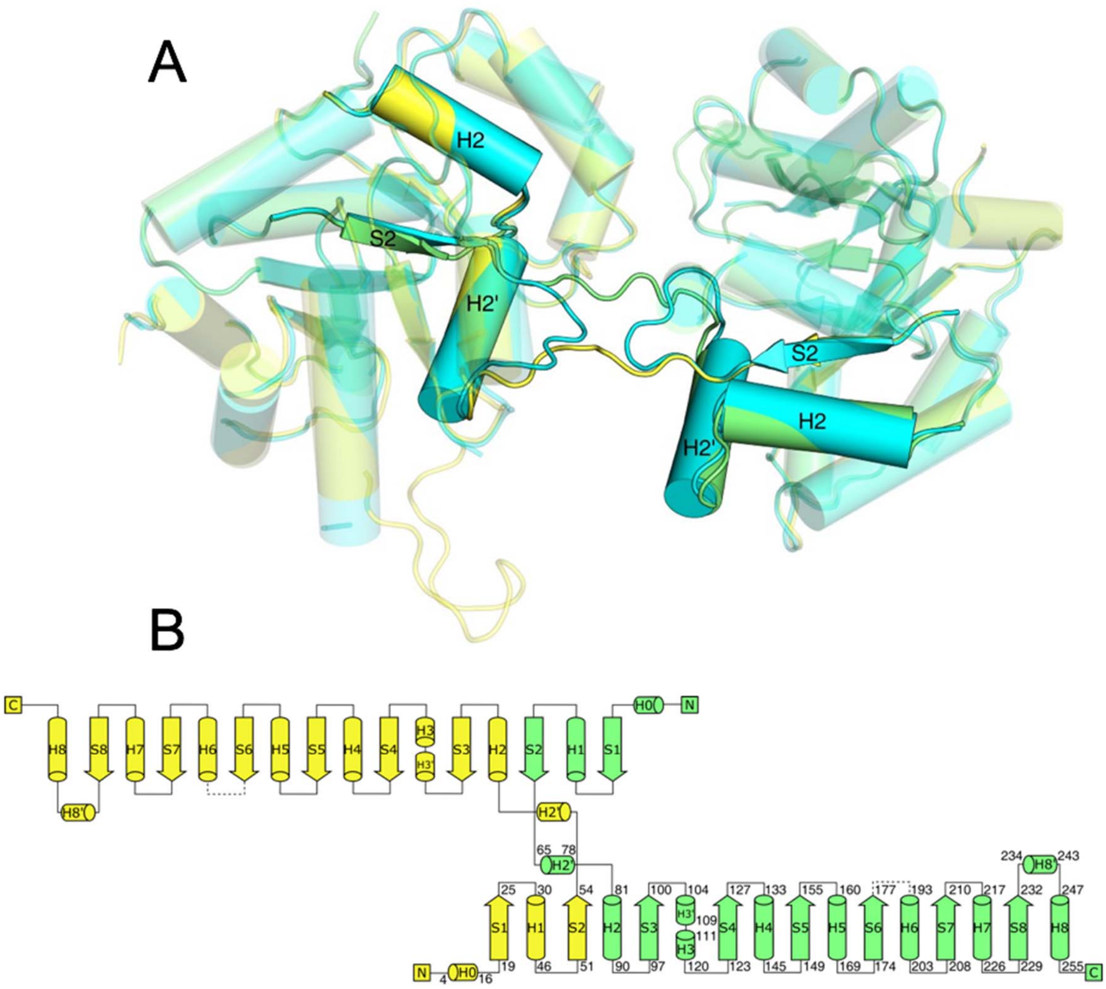

Figure S4 Illustration of the hinge region of the *Sp*TrpA 3D domain-swapped dimer CD (yellow-green), superposed on two copies of chain A from the *Sp*TrpAB structure (PDB id: 5KIN) (cyan). The β-strand S2 and helices H2' and H2 are labeled. In the *Sp*TrpAB structure, the linker (corresponding to the closed conformation) after emerging from strand βS2, first turns away from helix H2, then turns down, to finally turn again and enter helix H2'. In the open conformation, the linker continues directly from βS2 to helix H2', avoiding any crossing with its replica in the dimer. (A) A cartoon model; (B) a topology diagram.

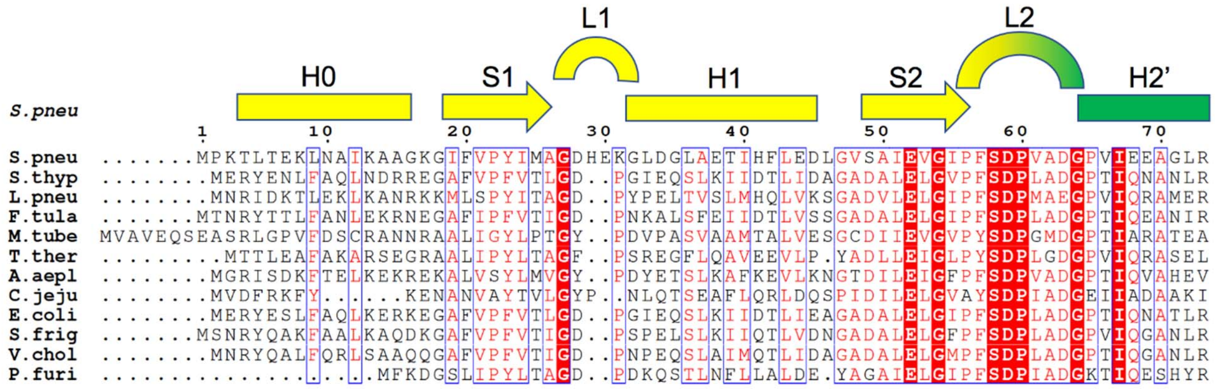

Figure S5 Structure-based sequence alignment of the N-terminal region of TrpA proteins with known crystal structures. The PDB deposits used in the alignments are as follow: 5KIN_C (S.pneu), 5CGQ_A (S.thyp), 50CW_W (M.tube), 1WQ5_B (E.coli), 5KMY_A (L.pneu), 3VND_H (S.frig), 3THA_B (C.jeju), 2EKC_B (A.aepl), 3NAV_B (V.chol), PE0K_K (P.furi), 5KZM_A (F.tula) and 1WXJ_A (T.ther). The surface protrusion helix H2’ is a topological novelty in TrpA that makes the S2-H2’ L2 loop a hinge suitable for 3D domain swapping. In addition, the *Sp*TrpA has a small insertion (L1) between S1 and H1 that makes domain swapping much more likely. This insertion is present only in the TrpA sequences from *Streptococcus, Lactococcus* and *Floricoccus* species. Yellow-highlighted secondary structure elements correspond to the N-terminal region that is swapped in the dimer, green corresponds to the C-terminal region. In the sequence alignment the red-highlighted amino acid residues are strictly conserved, red character amino acids are similar in a group and blue boxes indicate similarity across groups.

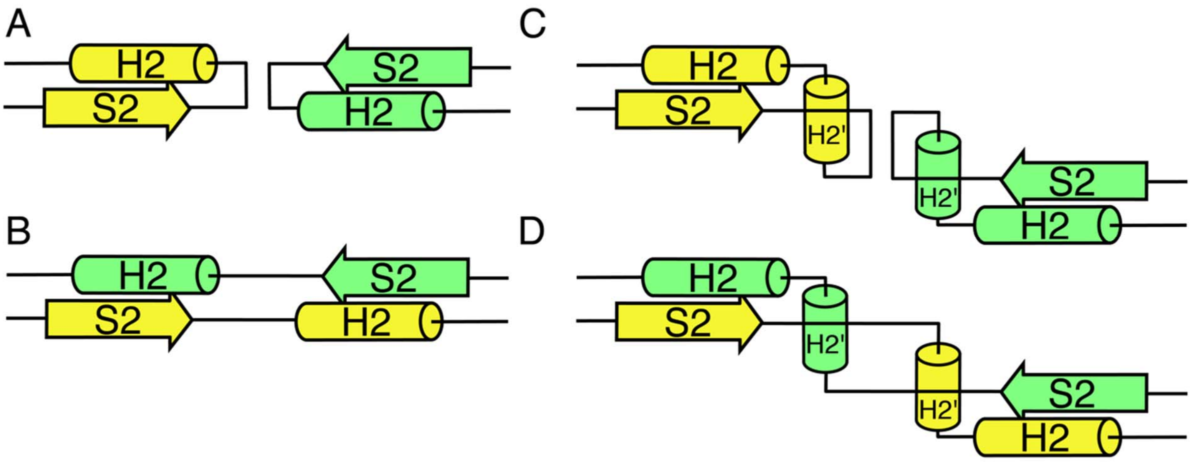

Figure S6 Comparison of a simple 3D domain swapping event (A, B), where the hinge loop simply has to straighten up, with a more convoluted change (C, D), as in *Sp*TrpA, that involves an intermediate helix (H2') element that helps to avoid direct linker intersection.
